# Developments in Trapped Ion Mobility Mass Spectrometry to Probe the Early Stages of Peptide Aggregation

**DOI:** 10.1021/jasms.2c00253

**Published:** 2023-01-12

**Authors:** Agathe Depraz Depland, Iuliia Stroganova, Christopher A. Wootton, Anouk M. Rijs

**Affiliations:** † Division of Bioanalytical Chemistry, Amsterdam Institute of Molecular and Life Sciences, Vrije Universiteit Amsterdam, De Boelelaan 1105, 1081 HV Amsterdam, The Netherlands; ‡ Bruker Daltonics GmbH & Co KG, Fahrenheitstraße 4, 28359 Bremen, Germany

## Abstract

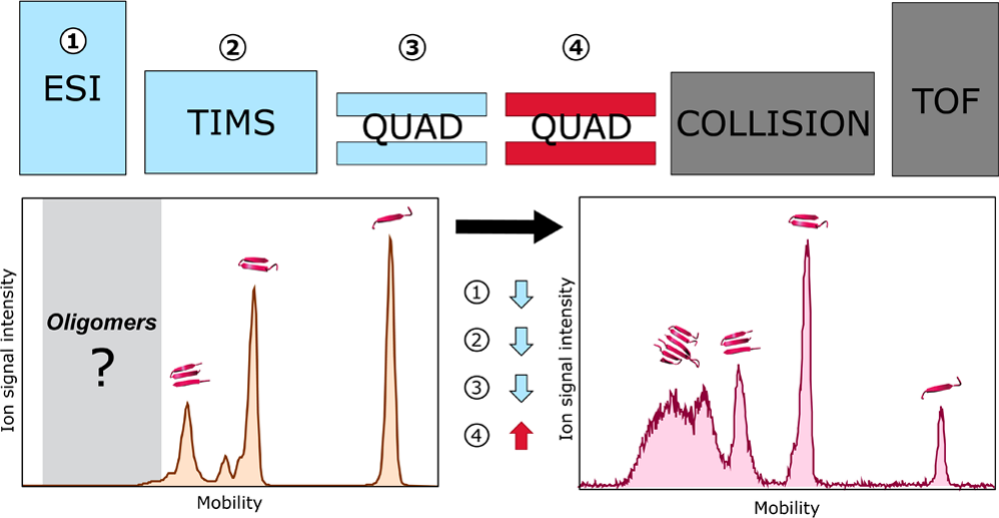

Ion mobility mass spectrometry (IM-MS) has proven to be an excellent method to characterize the structure of amyloidogenic protein and peptide aggregates, which are formed in coincidence with the development of neurodegenerative diseases. However, it remains a challenge to obtain detailed structural information on all conformational intermediates, originating from the early onset of those pathologies, due to their complex and heterogeneous environment. One way to enhance the insights and the identification of these early stage oligomers is by employing high resolution ion mobility mass spectrometry experiments. This would allow us to enhance the mobility separation and MS characterization. Trapped ion mobility spectrometry (TIMS) is an ion mobility technique known for its inherently high resolution and has successfully been applied to the analysis of protein conformations among others. To obtain conformational information on fragile peptide aggregates, the instrumental parameters of the TIMS-Quadrupole-Time-of-Flight mass spectrometer (TIMS-qToF-MS) have to be optimized to allow the study of intact aggregates and ensure their transmission toward the detector. Here, we investigate the suitability and application of TIMS to probe the aggregation process, targeting the well-characterized M307-N319 peptide segment of the TDP-43 protein, which is involved in the development of amyotrophic lateral sclerosis. By studying the influence of key parameters over the full mass spectrometer, such as source temperature, applied voltages or RFs among others, we demonstrate that by using an optimized instrumental method TIMS can be used to probe peptide aggregation.

## Introduction

Aggregation of proteins in the brain cells has been known to be responsible for the development of neurodegenerative diseases for over a century.^1^ To our current knowledge, over 50 proteins have been identified and correlated to the growth of amyloid fibrils in the neurons.^2,3^ Even though the full aggregation process remains far from fully understood, past studies have shown a recurring pattern in the final fibrillar structure^4^ as well as in the toxicity arising from the oligomeric species^5−7^ created at the early onset of the diseases. While the responsible protein is specific to each disease, such as the Amyloid β and Tau proteins for Alzheimer’s disease,^6,8−10^ the α-synuclein protein for Parkinson’s disease^11^ and the SOD1 and TDP-43 proteins in the case of amyloid lateral sclerosis (ALS),^12^ the process leading to the formation of fibrils seems to follow three phases in each case: a lag, an intermediary, and a saturation phase.^5,13,14^ A fundamental interest has been focused on the study of those amyloid structures, but the understanding of the intermediate structures present remains elusive. Typical studies to reveal the aggregation mechanism only allowed the characterization of the structure of the insoluble fibrillary assemblies or would give an averaged overview of the composition and shapes of the transient oligomers.^10,15−21^

A large panel of analytical techniques have been employed to probe amyloid fibril formation.^22^ For instance, structural techniques, such as X-ray diffraction, solid state Nuclear Magnetic Resonance (ss-NMR), or cryo-Electron Microscopy (cryo-EM), can be used on the late stages of aggregation to determine the structure of amyloid fibrils at high resolution.^4,8,22−26^ Techniques such as Fourier Transform InfraRed spectroscopy (FTIR), Circular Dichroism (CD), 2-Dimensionnal InfraRed spectroscopy (2D-IR), and Atomic Force Microscopy (AFM) bring valuable structural information over the ensemble present; however, they do not allow us to probe the early aggregation steps.^27−33^ Fluorescence Resonance Energy Transfer (FRET)^34,35^ or single molecule force spectroscopy^36^ can provide information about the molecular binding of proteins and protein assemblies. Separation methods have been used as well; when techniques such as Capillary Electrophoresis (CE), Size Exclusion Chromatography (SEC), and Analytical Ultra-Centrifugation (AUC) are combined with Dynamic Light Scattering (DLS) or Small-Angle X-ray Scattering (SAXS),^23^ the size and dynamic characteristics of separated oligomers present in solution can be determined.^22^ All these approaches either provide structural information on the resulting fibrillar stage or an averaged view of the aggregation process; however, they do not allow us to determine the structure of transient oligomers.

Ion Mobility Mass Spectrometry (IM-MS) has proven to be a powerful method for the analysis of *m*/*z* selected ions within a heterogeneous mixture of low concentrated analytes. With IM-MS, the mobility of charged molecules is measured, allowing the separation of these ions based on their size, mass, and charge, which is related to the overall 3-dimensional structure of the ion.^37−43^ For example, the addition of ion mobility to mass spectrometry allows one to separate and identify isomeric compounds^44−51^ or to unravel the conformational landscape of biomolecular assemblies.^52−59^ These IM-MS capabilities make it an ideal method to unravel the pathways of amyloid formation and to obtain oligomeric separation between charge states with similar *m*/*z* value. As of today, several ion mobility-based approaches have been developed, and the most common ones are Drift Tube (DTIMS), Differential (DIMS), Field Asymmetric (FAIMS), Traveling Wave (TWIMS), and more recently, the focus of this work, Trapped Ion Mobility Spectrometry (TIMS).^49,60−62^

Various IM-MS studies focus on protein and peptide aggregates, highlighting the possibilities to use IM-MS to determine the structure of those assemblies^58,63−66^ to characterize their formation^65,67,68^ or even monitor their dynamics.^69−73^ Moreover, IM-MS can also be applied to probe the interaction with molecules that influence the aggregation process,^74−80^ despite the nature of the transient oligomeric phase. Even though these experiments have brought more insight in the aggregation processes that coincide with the development of the neurodegenerative diseases, many important details on the exact sequence of these events remain still unclear.

High-resolution ion mobility mass spectrometry can help to identify the aggregation signatures from the oligomers formed during the early aggregation stage. However, when it comes to complex and commercial instruments such as the here used TIMS-qToF instrument (Bruker Daltonics), standard operating conditions can easily alter the nature of the analyzed ions.^81,82^ When traveling through the multiple components of the ion mobility mass spectrometer, the internal vibrational energy of the analyte ions can significantly increase, inducing ion heating and possible dissociation of the assemblies before reaching the detector. Ion heating is often observed in both MS experiments^83,84^ as well as in IM-MS^85−87^ experiments, when so-called “low-field” conditions are not matched. Recently, studies on ion heating have been extended to noncovalently bound assemblies,^88^ demonstrating that these ions can remain intact during the mobility separation but can fragment once they pass the mobility component of the instrument. This implies that while measuring the mobility of intact analytes, fragments reach the detector. This will partly result in a mismatch between the mobility and *m*/*z* values of these assemblies. Moreover, the measured mobility spectrum, extracted from a selected *m*/*z*, might display extra mobility peaks originating from larger complexes that fragment into the selected *m*/*z* channel. This phenomenon is responsible for the appearance of spurious ions as was observed in traveling wave^89^ or drift tube systems.^90,91^ Trapped ion mobility coupled to MS (TIMS) showed a potential to preserve the intact noncovalent assemblies of peptides when operated under “soft” instrumental conditions.^85,88^

In this paper, we investigate the suitability and application of trapped ion mobility mass spectrometry (TIMS) to probe the aggregation signatures of the well-studied TDP-43_307–319_ wild type segment (WT). This segment, and mutations of this segment, have been studied in detail by Laos et al. using a drift tube ion mobility coupled to MS as well as several other techniques.^30^ In these studies, they characterized the development of TDP-43 peptide assemblies within a time span of a few hours, revealing the formation of oligomers ranging from monomer to octamer for the WT peptide. The higher oligomeric region of the mobility spectra was not fully resolved due to the limited resolution of the used instrument. With this study, we aim to determine whether we can observe these higher order oligomers more clearly and diagnostically when using a system with higher resolving power such as the TIMS-qToF.

The use of the TIMS for aggregation studies is not straightforward, the experimental parameters have to be adjusted in order to develop a workflow that is more adapted to preserve the formed oligomers. The literature discusses the differences between soft and harsh operational conditions,^88,92,93^ indicating which alternative instrument parameters can be explored to study the aggregation process. In the TIMS spectrometer, the ions traverse multiple ion funnels, a first quadrupole ion guide that will be referred to as “multipole” in the following sections ion guide, a second quadrupole, and a collision cell after separation in the TIMS cell, all using different pressure regimes and electric field magnitudes.^94−99^ Here, we will focus on key settings and discuss their contributions to the observed ion mobility spectra of the aggregating TDP-43 wild-type segment to define a new standard way to operate the TIMS spectrometer to study the fragile molecular assemblies.

## Experimental Section

### Sample Preparation

The wild-type (WT) 307–319 segment of the TDP_43_ protein (TDP-43_307–319_) with sequence H_2_N-^307^MGGGMNFGAFSIN^319^-C=ONH_2_ was purchased from Biomatik and used without further purification (>98%). Ammonium acetate (AA) solution 5 M in H_2_O and ammonia solution 25% were purchased from Sigma-Aldrich. Initially, 1.5 mg of the WT peptide was dissolved in 0.8 mL of 1,1,1,3,3,3-hexafluoro-2-propanol (HFIP, LC–MS grade, > 99.8%, Sigma-Aldrich) and sonicated for 5 min to ensure total dissolution of the sample. This solution was then divided in 50 μL aliquots that were partially covered and left to dry at room temperature for approximately 15 h. Once the HFIP was fully evaporated, the aliquots were stored at −20 °C. A stock solution of 100 μM was prepared using 10 mM ammonium acetate (AA) buffer with pH 7.4 and 2% of HFIP. The pH of the buffer was adjusted to pH 7.4 using a 0.5% ammonia:water solution of pH 11.4. The stock solution was further diluted using the same AA buffer to a final concentration of 20 μM to be used in the TIMS-qToF for analysis. The WT samples were stored at room temperature and were analyzed after 1–7 days of their preparation.

### Ion-Mobility Mass Spectrometry: Experimental Details and Instrument settings

**Figure 1:**
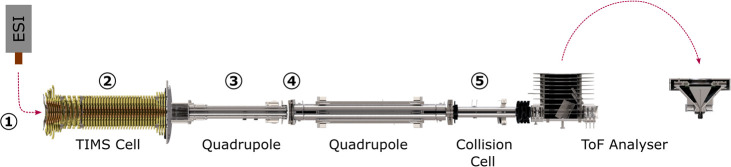
Schematic representation of the Bruker TIMS-qToF. The numbers indicate key regions and voltages that have been explored to develop the optimized method in order to study peptide aggregation.

Experiments were performed using a TIMS-qToF (first generation) instrument by Bruker Daltonics, which is described in more detail elsewhere.^93,95,100,101^ A schematic representation of the used TIMS mass spectrometer is presented in Figure 1. All measurements were acquired for 10 min using positive mode with electrospray ionization (ESI) via direct infusion using a flow rate of 120 μL·h^–1^. Two different sets of tuning parameters, named the Standard method and Optimized method, were used in this study, and their specific parameters are described in Table S1 of the Supporting Information. Additionally, key parameters, relevant for studying peptide aggregation, are summarized in Table 1. The gas pressure inside the TIMS tunnel for the standard method was left at its default value, 2.635 mbar, and was shifted to 2.404 mbar for the optimized method to be able to observe all ions mobilities within the set range. Using the ion mobility separation, species of a specific *m*/*z* values (*m* = mass, *z* = charge) that contain different [*n*]^+*z*^ (*n* = oligomer number) were separated by measuring their inversed reduced mobilities (1/*K*_0_). For simplification purposes, 1/*K*_0_ will be referred to as “mobility value” in the rest of this manuscript.

**Table 1:** Key Parameters for Both the Standard and the Optimized Method, Ordered According to Their Location in the TIMS Mass Spectrometer as Displayed in Figure 1

Figure 1	Parameters	Standard method	Optimized method
Source			
1	Temperature (°C)	200	50
MS			
3	Funnel 2 RF (Vpp)	500	100
3	Multipole RF (Vpp)	400	50
4	Ion energy (eV)	5	10
TIMS			
2	Δ6 (V)	100	38

### Ion Mobility and Mass Calibration

Agilent ESI tuning mix for ESI-ToF (*m*/*z* 322, 622, 922, 1222, 1522, 1822) was used to calibrate the reduced ion mobility spectra. The instrument was calibrated before every measurement using the standard set of parameters and following the acquisition. However, when the gas pressure inside the TIMS tunnel was altered, corresponding to the optimized set of parameters, preacquisition calibration for the mobility was not possible. Each spectrum acquired with similar operating settings was calibrated identically. First, an internal calibration of the tuning mix spectrum was performed using the following inversed reduced ion mobilities values, recommended by Bruker: 0.732, 0.985, 1.190, 1.382, 1.556, and 1.729 V·s·cm^–2^ (respectively with the mass previously given). Then, an external calibration was performed; i.e., the internal calibration described previously was applied on the spectra of the samples recorded with similar TIMS tunnel pressure.

## Results

### Standard vs Aggregation Method: How to Observe Aggregation with the TIMS

**Figure 2:**
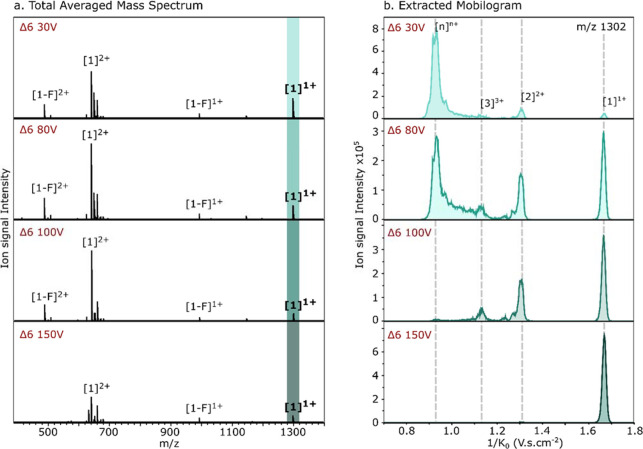
(a) Total averaged mass spectra of TDP-43_307–319_ WT peptide segment acquired with the standard method (top) and the optimized method for peptide aggregation (bottom). (b) Extracted ion mobilogram recorded at *m*/*z* 1301.5 (± 0.01) corresponding to the monomeric ion of mass [M + H]^1+^ for the standard (top) and optimized method (bottom); the asterisk highlights the presence of spurious ions. (c) The zoom-in isotopic distribution of the mass spectrum extracted from the mobilogram peaks acquired with the optimized method. It displays four different distributions for the following reduced mobility values, from top to bottom: 1/*K*_0_ = 1.67; 1.32; 1.14 and 1.05 V·s·cm^–2^ with Δ*m*/*z* 1.0, 0.5, 0.33, and 0.25, respectively. The gray isotopes peaks displayed in the bottom spectra show participation of fragmented peptide aggregates from the triply charged trimer.

In order to study the aggregation signatures of the WT peptide using trapped ion mobility mass spectrometry, the instrumental parameters for ion mobility have to be evaluated. Figure 2a shows the averaged mass spectrum using the standard method (i.e., same set of parameters used for the calibration of the instrument, described as optimal to observe the *m*/*z* and mobility in a range of values including the *m*/*z* of our target analyte of *m*/*z* 1301.5) and the optimized method for aggregation, whose parameters are described in Table 1 and in Table S1 of the Supporting Information. Both MS spectra display identical peaks at *m*/*z* of 1301.5, 651.3, 999.5 and 500.2, corresponding to the singly and doubly charged monomeric ion ([M + H]^1+^ and [M + 2H]^2+^) and their dominant fragments ([1 – F]^1+^ and [1 – F]^2+^) resulting from b/y fragmentation at the b_4_/y_9_ peptide bond. Figure 2b presents the extracted mobilograms at *m*/*z* 1301.5 for both methods. In the top reduced ion mobility spectrum (i.e., mobilogram), measured with the standard method, three main peaks are observed. The most intense, at 1/*K*_0_ 1.67 V·s·cm^–2^, originates from the mobility of the monomer singly charged ([1]^1+^). The consecutive two other intense peaks, at 1/*K*_0_ 1.32 and 1.14 V·s·cm^–2^, originate from the doubly charged dimer [2]^2+^ and the triply charged trimer [3]^3+^, respectively. Larger ions can fragment into smaller assemblies, and therefore, additional peaks in the mass spectrum can appear with a different isotopic distribution, as illustrated in Figure 2c (bottom spectrum, gray peaks showing the presence of the triply charged trimer). The assignment of the ion mobility peaks was done by using the extracted mass spectra of each reduced ion mobility peak and evaluating the observed isotopic distribution, as depicted in Figure 2c. A comparison with similar experiments performed on a drift tube ion mobility mass spectrometer by Laos et al. show the improved resolution when using the TIMS-qToF instrument. This is further discussed in Figure S4 of the Supporting Information.

The extracted mobilogram (orange) obtained with the standard method shows a clear peak at 1/*K*_0_ 1.24 V·s·cm^–2^, which corresponds to a spurious ion that most probably results from the dissociation of a larger, multiply charged precursor ion. The bottom mobilogram (pink), measured with the optimized method, does not display this spurious ion, and moreover, features at lower reduced mobility values are favored. This coincides with a lower relative abundance of the [1]^1+^, [2]^2+^, and [3]^3+^ species already present in the mobilogram acquired with the standard method. The additional observed peaks comprise of a band at 1/*K*_0_ 1.05 V·s·cm^–2^ corresponding to [4]^4+^ oligomers mixed with other spurious ions, as can be seen from the isotopic distribution of this band Figure 2c. The distribution of peaks between 1/*K*_0_ 1.0 and 0.8 V·s·cm^–2^ potentially originates from the presence of higher order oligomers.

While the mass spectra of the two different instrumental methods show the same *m*/*z* species, the extracted ion mobilograms are clearly different. The optimized method allows us to observe a more accurate distribution of the higher order oligomers, with an increased signal intensity and resolution at the lowest reduced ion mobility values. Additionally, the presence of spurious ions, such as, for example, the peak observed at 1/*K*_0_ 1.24 V·s·cm^–2^ in Figure 2b (asterisk, top, orange mobilogram), originating from the dissociation of peptides assemblies on their path toward the MS detector, is mostly avoided. In the sections below, key parameters of the optimized method and their influence on probing the oligomers have been identified and discussed in the order of appearance in the TIMS spectrometer, which are indicated by the numbers 1–4 in Figure 1.

### Influence of the Source Temperature

**Figure 3:**
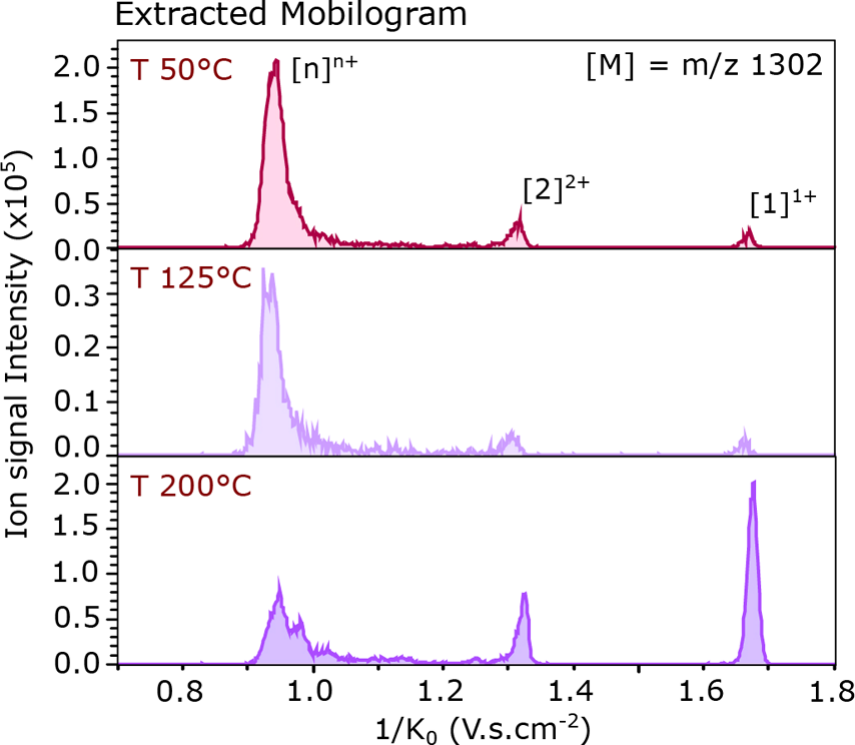
Extracted ion mobilograms displaying the reduced mobility recorded for the *m*/*z* 1301.5 (± 0.01) of the TDP-43_307–319_ WT sample. The temperature (*T*) of the source was increased in steps of 75 °C between each acquisition from top to bottom spectra.

The source temperature, indicated by 1 in Figure 1, affects both the desolvation of the analytes as well as the nature of the ions entering the mass spectrometer. We have studied the influence of the source temperature on the appearance of peptide oligomers of WT of TDP-43_307–319_. Under standard operation conditions, the temperature is set to about 200 °C. In this study, we decreased the temperature to 50 °C, investigating the mobilogram at 200, 125, and 50 °C. Figure 3 shows the extracted reduced ion mobility spectra of the *m*/*z* 1301.5 recorded using the standard instrument parameters with a variable source temperature.

When the temperature is lowered, peaks at lower reduced mobilities are favored. At 50 °C, the mobilogram clearly shows three peaks. The first peak (from the right) at 1/*K*_0_ = 1.68 V·s·cm^–2^ corresponds to the monomer singly charged, the second peak at 1/*K*_0_ = 1.32 V·s·cm^–2^ is the dimer doubly charged, and a last peak, the most intense one, at 1/*K*_0_ = 0.95 V·s·cm^–2^ seems to indicate the presence of larger oligomers (>4 units). With an increase of 75 °C (*T* = 125 °C), the peak distribution is conserved, but with a lower signal-to-noise ratio. Once the temperature is increased to 150 °C or higher, the intensity ratio is reversed compared to the mobilogram acquired at 50 °C: the monomer ([1]^1+^) becomes dominant, while higher oligomeric states (>[2]^2+^) decrease significantly in intensity.

The TIMS resolution at 200 °C seems better, resulting in a sharper monomeric peak. Additionally, multiple peaks are present in the oligomeric region, which are, at this moment, unresolved and masked in the tail of the peak at 1/*K*_0_ = 0.95 V·s·cm^–2^ at lower temperatures. These peaks originate from oligomers as well as spurious ions that dissociated into monomeric units, as mentioned in the previous section. In the case of noncovalently bound peptide assemblies, a lower temperature is preferred to preserve present higher order oligomers, while the mobility resolution can be improved with the settings discussed below. Additionally, it helps to control the increase of internal energy of the created ions.

### Influence of the Delta 6 Potential of the TIMS Cell

After ionization, the ions are transferred into the TIMS cell, indicated by number 2 in Figure 1, which combines five distinctive parts, where different voltages and RF fields can be applied (for details see Figure S1 of the Supporting Information). The TIMS cell is comprised of the entrance funnel to focus the ions entering the cell, the first tunnel, where ions get accumulated to improve the sensitivity, the second tunnel to separate the ions according to their CCS value (named ramp in Figure S1), the “gate” between those two tunnels (transfer in Figure S1), and an exit funnel, where the ions will elute from, one after the other to travel onward in the rest of the mass spectrometer (exit in Figure S1). As mentioned before, the ions encounter different potentials, named delta 1–6 (Δ1–6),^102−104^ as they travel through these different regions, which will affect the way that the ions will be separated and subsequently transmitted to the MS detector.

**Figure 4:**
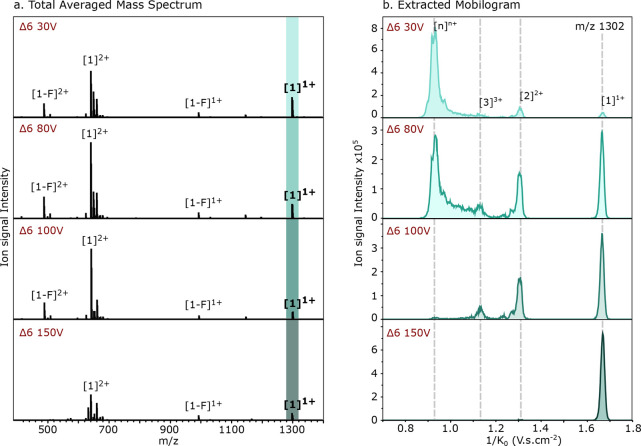
Influence of the Delta 6 (Δ6) potential over the transmitted ions through the TIMS-ToF from the WT TDP-43_307–319_ peptide: (a) total averaged mass spectra; (b) extracted reduced mobility spectra of the *m*/*z* 1301.5 (± 0.01).

To conserve and measure the formed peptide oligomers, the delta 6 (Δ6) has proven to be critical. This potential is used for the transfer of the ions from the accumulation tunnel exit to the separation tunnel, where the electric field gradient is applied and slowly ramped down at a chosen rate to separate the ions of different mobility. Since this voltage is applied over a short distance (approximately 1 cm), the resulting electric field can be quite strong. In order to conserve the peptide aggregates, we slowly ramped down the Δ6 potential to determine its optimal value, i.e., where maximum mobility separation was achieved while keeping the oligomers intact. In the case of the commercial TIMS-qTOF used for these experiments, the Δ6 parameter is intrinsically linked to the mobility range set on the instrument and, therefore, on the pressure inside the TIMS cell. In order to keep the pressure sufficient to ensure the separation, and at the same time a range of mobility as wide as necessary to see the full display of peptide assemblies of the same *m*/*z* value, the lowest possible D6 achievable was 38 V. Figure 4a presents the total mass spectrum of WT of the TDP-43_307–319_ segment with an increased Δ6 value from 30 to 150 V (from top to bottom), and Figure 4b shows the corresponding extracted reduced ion mobility spectra for *m*/*z* 1301.5.

As observed for the source temperature, the ion mobility distribution drastically changes with the Δ6 values, while the mass distribution is only mildly affected in its signal intensity. Lowering the Δ6 potential results in the appearance of clear peaks at the lower reduced ion mobility values (1/*K*_0_), which are more intense than the peaks originating from the monomer ([1]^1+^) and dimer ([2]^2+^). This indicates that formed higher order oligomer ions ([*n*]^*nz*+^) seem to fragment between the accumulation and the separation phase in the TIMS cell when too high Δ6 potentials are applied. Therefore, the obtained reduced ion mobility spectra will not display a fair representation of what is initially infused in the mass spectrometer. The optimal value to study aggregates has been defined as 38 V, as at this voltage the oligomers present in solution were visualized with the best signal intensity and resolution of the mobilities.

### Influence of the RF Amplitudes between the Mobility Cell and the Mass Spectrometer

Next, the influence of a set of radio frequencies (RF1/RF2), indicated by 3 in Figure 1, on the observed reduced ion mobility distributions of the mass *m*/*z* 1301.5 from the WT TDP-43_307–319_ peptide has been studied. RF1 is applied on each component of the TIMS cell and RF2 on the multipole located right after the exit funnel. From the standard set of parameters, we ramped down the amplitude of these RFs (RF1/RF2) from 500/400 to 300/200 to 100/50 Vpp. Figure S2 of the Supporting Information shows the resulting reduced mobilograms for each set. Altering the RF voltages does not significantly improve the transmission of smaller oligomer ions. The peaks in the mobilogram (Figure S2) observed at 1/*K*_0_ = 1.68 and 1.32 V·s·cm^–2^, which originate from [1]^1+^ and [2]^2+^, respectively, preserve a similar relative abundance. However, the contribution from the higher order oligomers at 1/*K*_0_ = 0.95 V·s·cm^–2^ has increased in intensity when the RF voltages are minimized. Using a lower RF setting, namely 100/50 Vpp, results in an improved transmission of the higher charge states oligomers, while the transmission of the lower charge states remains mostly unaffected.

### Influence of the Quadrupole Entrance Lens Energy: Ion Energy (IE)

**Figure 5:**
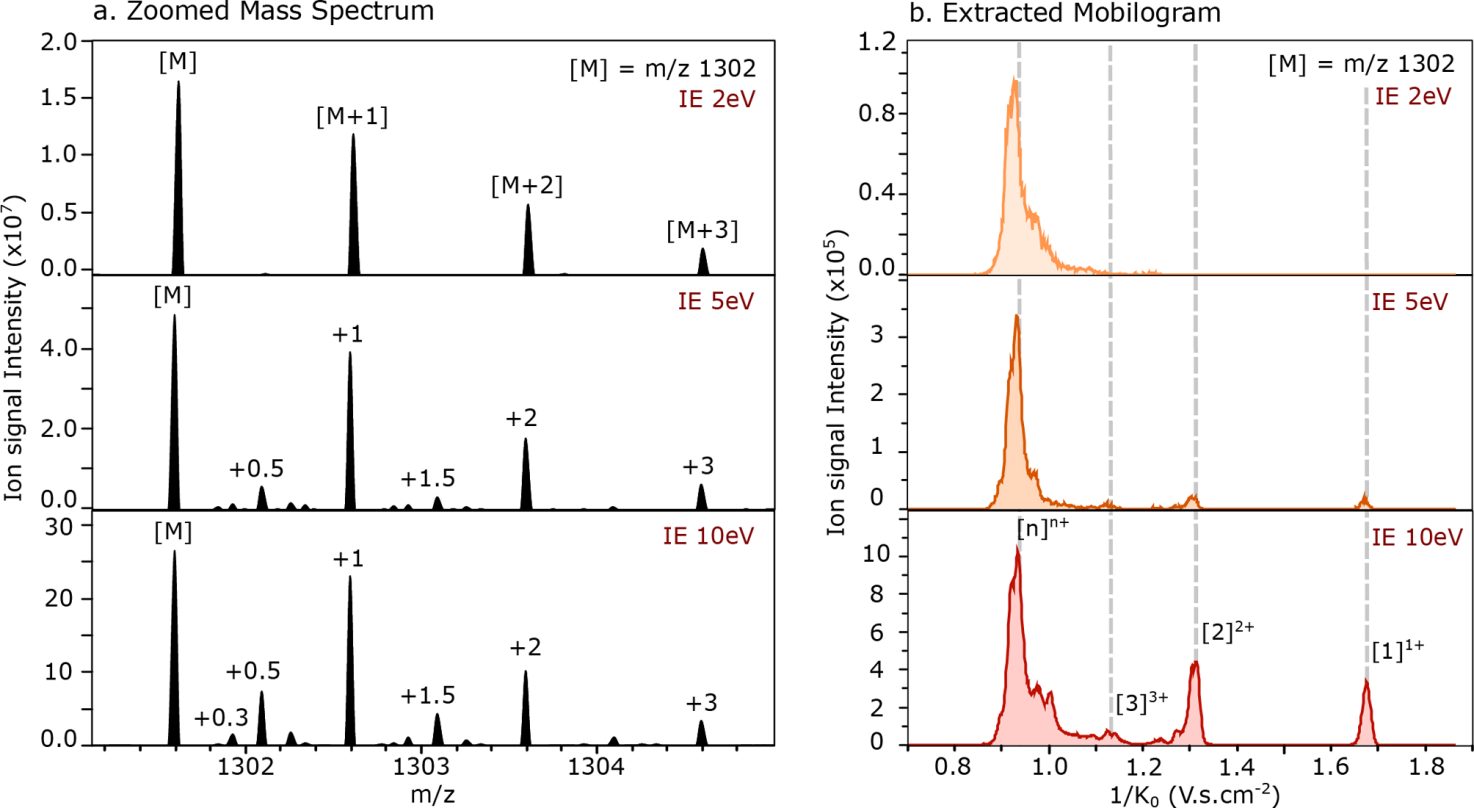
Influence of the ion energy on the transmission of the higher order oligomers from the TDP-43_307–319_ WT peptide. (a) Mass spectra zoomed-in on the *m*/*z* 1301.5 region showing the isotopic distribution of the present [*n*]^*nz*+^ ions at three different ion energies. The value of each spacing, between [M] = *m*/*z* 1301.5 and the isotope peaks, is indicated on the top of each *m*/*z* peak. (b) Extracted reduced ion mobility of the *m*/*z* 1301.5 peak (± 0.01) for the same ion energy values.

The ion energy, indicated with 4 in Figure 1, is the bias voltage between the multipole and the end of the quadrupole to allow the transmission of the ions toward the collision cell. Typically, the ratio between IE and collision cell energy (CE) is set about 2:1, where the total sum of IE and CE gives the decrease of the voltages from the multipole to the collision cell region. The influence of the ion energy over the nature of the transmitted ions is investigated by monitoring changes in both the mass spectrum as well as in the mobilogram. The isotopic distribution in the mass spectra presented in Figure 5a displays a wider charge distribution as the ion energy is increased from 2 to 10 eV. At 2 eV, only the isotope distribution of singly charged monomers is observed (with Δ*m*/*z* 1) when zooming on the *m*/*z* channel of the [*n*]^*nz*+^ ions. With increasing ion energies, to 5 eV, a wider range of charge distribution is observed with peaks appearing with Δ*m*/*z* 0.5, 0.33, and 0.25 corresponding to the presence of [2]^2+^, [3]^3+^ and [4]^4+^, respectively, is observed in the mass spectrum. At 10 eV, the isotopic distribution is similar as the one measured at 5 eV, but the intensity of the peaks corresponding to the higher charge state oligomers increases.

The corresponding reduced ion mobility spectra (see Figure 5b) look contradictory at first sight with the presence of a large peak at 1/*K*_0_ = 0.95 V·s·cm^–2^ corresponding to higher charge state aggregates, although only a +1 isotopic distribution is observed in the mass spectrum with IE = 2 eV. When the ion energy is increased to 5 eV, the reduced mobility signal of the low charge states, between 1/*K*_0_ = 1.05 and 1.68 V·s·cm^–2^, gain in intensity. For the highest value of the ion energy (10 eV), numerous peaks are present in the extracted reduced mobility spectra of the *m*/*z* 1301.5 ion corresponding to the presence of the full oligomeric range, from [1]^1+^ to >[4]^4+^. The reduced mobility spectra show that with low ion energy the signal of lower charge state assemblies disappears, which indicates that when the ion energy is set too low, the ions are not transmitted. When the entrance lens of the quadrupole does not provide enough energy to the coming ions, those fail to reach the end of the quadrupole. When higher ion energy values are used, the isotopic distributions from the averaged mass spectra show that indeed the higher the charge state oligomers such as [3]^3+^ and [4]^4+^ are detected.

With the optimized instrumental method, higher order WT oligomers are separated in the mobility cell; however, at this moment no aggregates larger than [4]^4+^ are observed in the mass spectrum. This indicates that these larger oligomers dissociate between the TIMS cell and the detector. Their mobility is still measured, but these ions are associated with the mass of smaller assemblies, e.g., as the singly charged monomer as observed by the +1 isotopic distribution for IE = 2 eV (see the top panel of Figure 5a).

### Identification of the Ions with Mobility Measured in the Suspected Oligomeric Region

**Figure 6:**
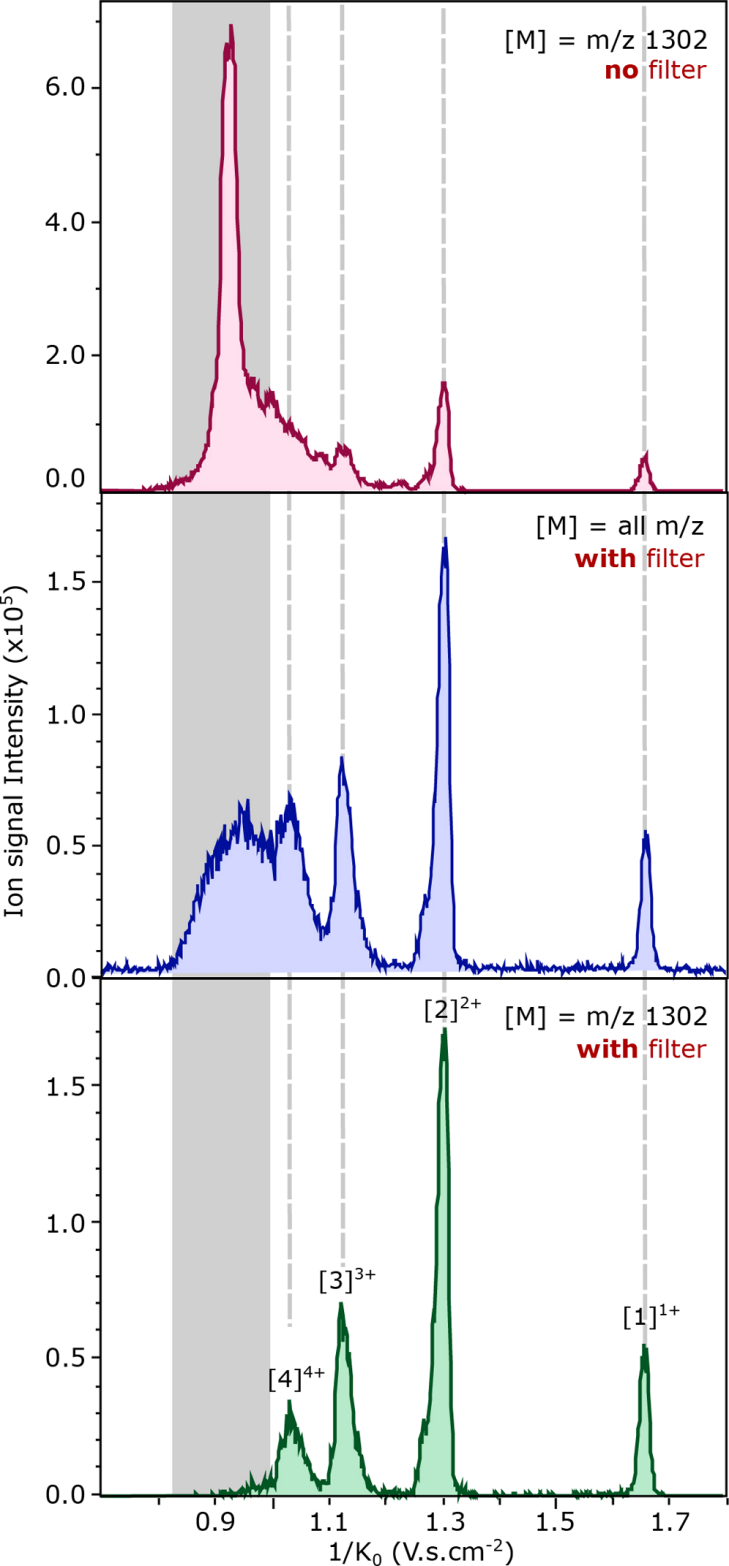
Influence of the quadrupole filtering on the transmission of the TDP-43_307–319_ WT peptide ions present in the higher order oligomer region. The top spectrum (pink) shows the extracted mobilogram of *m*/*z* 1301.5 without quadrupole filtering (full transmission mode), middle mobilogram (blue) all ions but with quadrupole filtering of the *m*/*z* 1301.5 ± 5 *m*/*z*, bottom (green) shows the extracted mobilogram of *m*/*z* 1301.5 to 1303.6 with quadrupole filtering of the *m*/*z* 1301.5 ± 5 *m*/*z*.

As discussed in the previous section, oligomeric ions can undergo dissociation in different regions of the instrument before reaching the detector. This can significantly complicate the interpretation of the data. Here, we investigate the exact origin of the intense peak present in the 1/*K*_0_ = 0.85 to 1.0 V·s·cm^–2^ range in the extracted mobilogram of the *m*/*z* 1301.5 channel; see Figure 6 (top panel, pink trace). This mobilogram, and the other two, are acquired using the aggregation optimized set of parameters as described in Table 1. The blue trace in Figure 6 (middle panel) shows the total mobility spectrum using the quadrupole filtering at *m*/*z* 1301.5 ± 5, therefore only transmitting the mass of interest through the quadrupole. This mobilogram displays four main peaks at 1/*K*_0_ = 1.68, 1.32, 1.14, and 1.04 V·s·cm^–2^, respectively, from [1]^1+^, [2]^2+^, [3]^3+^, and [4]^4+^ as indicated by the gray lines and a broad less resolved peak in the oligomeric region between 1/*K*_0_ = 0.85 and 1.0 V·s·cm^–2^. The extracted mobilogram (*m*/*z* 1301.5) including quadrupole filtering of the same *m*/*z* channel is presented in the lower panel of Figure 6 (green trace). This mobilogram shows similar peaks at 1/*K*_0_ = 1.68, 1.32, 1.14, and 1.04 V·s·cm^–2^ resulting from the [*n*]^*nz*+^ oligomers and a very small peak in the oligomeric region at 1/*K*_0_ = 0.98 V·s·cm^–2^.

The disappearance of this intense ion mobility signal peak in the oligomeric region between the not-quadrupole-filtered (pink) and the filtered mobilogram (green) indicates that their mass over charge ratio, after mobility separation, is different than the one set by the quadrupole (*m*/*z* 1301.5). When all ions are transmitted by the quadrupole, ions with low reduced mobility (1/K_0_= 0.85 to 1.0 V·s·cm^–2^) are detected in the *m*/*z* 1301.5 channel; however, most of these ions do not reach the detector when the quadrupole filter is enabled at *m*/*z* 1301.5. This peak in the pink mobilogram most likely originate from ions with larger *m*/*z* values that dissociate into the 1301.5 *m*/*z* channel between the quadrupole and the entrance of ToF detector. Although this suggest that the ions have a different origin than the expected [*n*]^*nz*+^ aggregates, the presence of higher charge states assemblies of the monomer units with mobility 1/*K*_0_ = 0.85–1.0 V·s·cm^–2^ is not discarded. The blue mobilogram, obtained with the quadrupole mass filter at 1301.5 ± 5 does show a broad peak in the oligomer region indicating the presence of [*n*]^*nz*+^ aggregates. However, due to fragmentation in the collisional cell region, they do not appear in the green mobilogram. This allows us to conclude that the dissociation—in the collision cell, and to a lesser extent in the multipole region—of larger oligomers into smaller monomeric units contributes largely to the ions observed with *m*/*z* 1301.5 and mobility 1/*K*_0_ = 0.85–1.0 V·s·cm^–2^ (oligomeric region) in the blue mobilogram.

**Figure 7:**
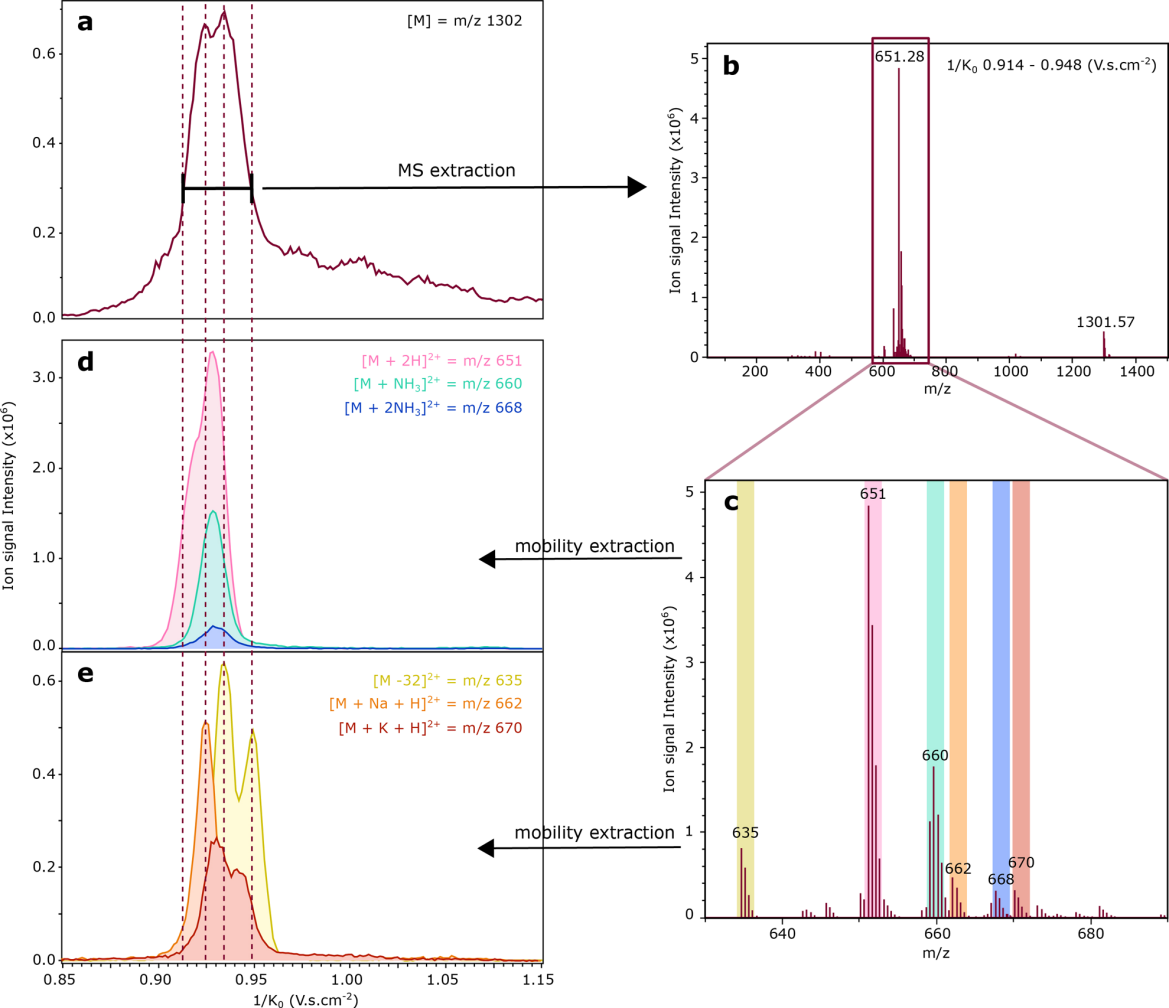
Identification of the ions undergoing dissociation after the mobility separation in the oligomer region from 0.914 to 0.948 1/K0. (a) Extracted mobilogram at *m*/*z* 1301.5 zoomed in on the region of interest. (b) Extracted MS spectrum from the peak in (a). (c) MS spectrum between *m*/*z* 630–690 highlighting the regions for the extracted mobilograms of d) and e). d) Extracted mobilogram of the *m*/*z* 651.3 corresponding to the monomer doubly charged (pink), in green the extracted mobilogram of the *m*/*z* 659.8 corresponding to the monomer doubly charged with added ammonia adduct, and in blue the extracted mobilogram of the *m*/*z* 667.8 corresponding to the monomer doubly charged with two added molecules of ammonia. e) Extracted mobilogram of the *m*/*z* 634.8 (yellow), in orange the extracted mobilogram of the *m*/*z* 662.3 corresponding to the monomer protonated with an adduct of sodium, and in red the extracted mobilogram of the *m*/*z* 670.3 corresponding to the monomer protonated with an adduct of potassium.

In order to assign the possible identity of the intense peak in this 1/*K*_0_ = 0.85–1.0 V·s·cm^–2^ range of the pink mobilogram, the mass spectrum over the corresponding mobility range has been extracted as illustrated Figure 7a,b. This mass spectrum indicates that besides the [*n*]^*nz*+^ peak at *m*/*z* 1301.5, numerous ions with *m*/*z* values between *m*/*z* 600–700 possess similar mobility values. We have extracted the mobilogram from each *m*/*z* peak as indicated in Figure 7c. Subsequently, the position of each extracted mobility peak is compared to the position of the original unidentified peak; see Figure 7d,e. Most ions falling in this *m*/*z* range have a inversed mobility of 1/*K*_0_ = 0.85 and 1.0 V·s·cm^–2^, such as the sodiated doubly charged monomer with *m*/*z* 662.3 at 1/*K*_0_ = 0.925 V·s·cm^–2^ (orange) and a doubly ion with *m*/*z* 634.8 at 1/*K*_0_ = 0.935 V.s.cm^–2^ (yellow). However, when these doubly charged ions are isolated with the quadrupole, none of these ions fragment into the *m*/*z* 1301.5 channel, even after adding significant collision energy (see Figure S3).

As discussed above, when studying peptide aggregation, ion heating and the resulting dissociation possibilities make the analysis significantly complex. Various ions with different *m*/*z* values, such as complexes or other higher order aggregates with a different *n*/*z* ratio (monomer to charge ratio), can end up in the studied *m*/*z* channel, while formed [*n*]*^nz+^* aggregates of the studied *m*/*z* appear in another *m*/*z* channel. Since ion heating can in principle occur at every stage, after the TIMS cell ions can undergo dissociation in the multipole, quadrupole and collision cell region. Theoretically, ions can fragment multiple times when they traverse from the TIMS cell to the ToF region, resulting then in both extra mobility and *m*/*z* peaks.

Here, the additional use of the quadrupole mass filter (blue and green trace Figure 6) can help to assign and simplify the recorded mobilograms by removing spurious ions. For example, besides the above discussion on the intense peak 0.92 V·s·cm^–2^, also the peaks at 1.22 and 1.09 and 1.0–0.95 V·s·cm^–2^ (pink trace), which originate from larger aggregates or complexes that fragment into the *m*/*z* 1301.5 channel between the quadrupole and the ToF entrance, disappear when quadrupole filtering mode is applied (green trace).

## Summary and Conclusions

In this paper, we have demonstrated the necessity to optimize the workflow when using trapped ion mobility mass spectrometry to study the fragile, noncovalently bound oligomers formed during the aggregation process. By investigating essential instrument parameters, we have shown that it is not straightforward to preserve oligomeric assemblies until they have reached the MS detector in the TIMS. Starting from the infusion in the spectrometer, parameters such as temperature, delta potentials, RF voltages, ion energy, have been explored and subsequently optimized to observe the formed early stage oligomers in the aggregation of the 307–319 segment of the TDP_43_ protein (TDP-43_307–319_). The parameters and workflow discussed in this work to study the aggregation of the TDP-43 peptide segment can be used to probe aggregation of segments from other proteins related to neurodegenerative diseases. Moreover, these settings can be translated when using other ion mobility mass spectrometers or can function as a starting point when studying other noncovalently bound complexes or analyte in the same molecular weight and charge range.

We identified the collision cell region of the mass spectrometer as being the main source of ion heating, inducing therefore dissociation of the ions after the mobility separation but before the mass detection. However, the multipole region also plays a role in the heating of the ions. Because of these processes, the signal emanating from the higher oligomeric species (>[4]^4+^) might appear in different *m*/*z* channels. Moreover, oligomer signatures are masked by the fragment signals from other ions with similar mobility values, such as larger and multiply charged assemblies and metal-ion complexes, making it challenging to probe aggregation pathways. Using quadrupole filtering, we have been able to both assign these contributions and to visualize formed oligomers. With the presented soft analysis conditions, we have demonstrated that trapped ion mobility mass spectrometry can be used to study aggregation processes by minimizing the ion heating and thereby prevent fragmentation of the formed oligomers. Lowering the ion heating process with even softer instrumental conditions strongly facilitates the comprehension of the recorded data. These developments make it possible to probe the temporal evolution of the peptide aggregation using the TIMS-qToF spectrometer. Moreover, the reported workflow and parameters can either be used to study other intermolecularly noncovalently bound complexes of similar molecular weight and charge or they can be translated to a set of parameters when peptide aggregation is studied using another type of IM-MS spectrometer with similar mobility and spectral resolution.

## Supporting Information Available

TIMS instrumental parameters for standard and aggregation optimized measurement methods (Table S1); schematic of the TIMS cell highlighting the Δ6 potential (Figure S1); influence of the RF amplitudes between the mobility cell and the mass spectrometer (Figure S2); MS/MS spectra of the *m*/*z* 651.3 ± 5 at three different collision energies (Figure S3); comparison between Drift Tube experiment from literature and TIMS experiment (Figure S4) (PDF)
