# Supplementary material for: Developments in Trapped Ion Mobility Mass Spectrometry to Probe the Early Stages of Peptide Aggregation
Source: J Am Soc Mass Spectrom. 2023 Jan 12;34(2):193–204. doi: 10.1021/jasms.2c00253 (PMC9896548; doi:10.1021/jasms.2c00253)
Supplement: Supplementary file 1 — js2c00253_si_001.pdf [file js2c00253_si_001.pdf]

Supporting Information

Developments in Trapped Ion Mobility Mass spectrometry to probe the early stages of peptide aggregation

Agathe Depraz Depland[1], Iuliia Stroganova[1], Christopher A. Wootton[2], and Anouk M. Rijs[1,*]

1. Division of Bioanalytical Chemistry, Amsterdam Institute of Molecular and Life Sciences, Vrije Universiteit Amsterdam, De Boelelaan 1105, 1081 HV Amsterdam, the Netherlands, Correspondence to: Anouk M. Rijs, e-mail: a.m.rijs@vu.nl

2. Bruker Daltonics GmbH & Co KG, Fahrenheitstraße 4, 28359, Bremen, Germany

Table of Contents

S1 TIMS measurements for TDP-43$_{307–319}$ wild type peptide (WT) 2

Table S1. TIMS instrumental parameters for standard and aggregation optimized measurement methods (see Figure 1 in the main text) 2

Figure S1. Schematic of the TIMS cell highlighting the Δ6 potential 3

Figure S2. Influence of the RFs amplitudes between the mobility cell and the mass spectrometer . 3

Figure S3. MS/MS spectra of the m/z 651.3 +/- 5 at three different collision energies 4

Figure S4. Comparison of mobility spectra from drift tube experiment from the literature and TIMS-ToF experiments of 1301.5 m/z. 5

S1 TIMS measurements for TDP-43$_{307–319}$ wild type peptide (WT)

Table S1. TIMS instrumental parameters for standard and aggregation optimized measurement methods (see Figure 1 in the main text)

	Parameters	Standard method	Optimised method
**Source**			
**1**	*Temperature (°C)*	200	50
	Capillary voltage (V)	3500	3000
	Dry gas (L/min)	3.5	4.0
	Nebulizer (Bar)	0.3	0.8
**MS**			
**3**	*Funnel 2 RF (Vpp)*	500	100
**3**	*Multipole RF (Vpp)*	400	50
**4**	*Ion energy (eV)*	5	10
	Collision energy (eV)	10	1
	Collision Cell RF (Vpp)	2000	1300
	IMS Collision cell In (V)	300	200
**TIMS**			
**2**	*Δ6 (V)*	100	38
	Δ1 (V)	-20	0
	Δ2 (V)	-150	0
	Δ3 (V)	70	20
	Δ4 (V)	100	40
	Δ5 (V)	0	0
	Accumulation time	10	5
	Ramp time (ms)	70	100
	Funnel 1 RF (Vpp)	350	250
	Tunnel Out Vacuum (mBar)	0.85	0.89
	Tunnel In Vacuum (mBar)	2.410	2.40

Table S1: Recapitulative table of the parameters for both the standard and the optimised method, numbered according to the location of the parameters displayed Figure 1. °C corresponds to temperature in in degree Celsius, Vpp corresponds to Volt peak to peak, eV corresponds to electron Volt, L/min corresponds to litre per minute, ms corresponds to milliseconds, mBar corresponds to milliBar, and V corresponds to Volts.

Figure S1. Schematic of the TIMS cell highlighting the Δ6 potential

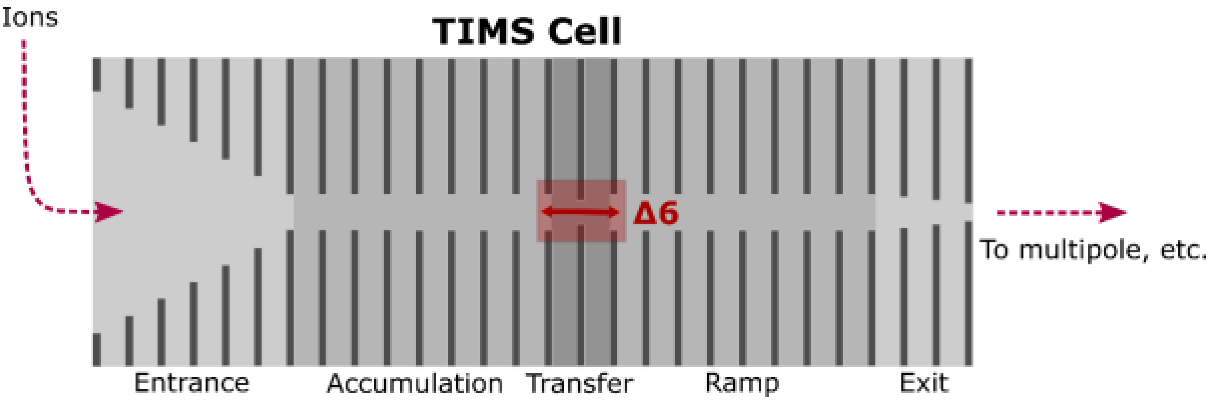

Figure S1: Schematic representation of the TIMS cell located between the ion source and the first quadrupole (multipole) of the mass spectrometer as indicated by number 2 in Fig.1. The pink doted arrows show the direction of the ion stream. The TIMS cell is divided in five distinct areas depicted here in different shades of grey. The Delta 6 (Δ6) potential is applied between the end of the accumulation tunnel and the beginning of the separation tunnel where the electric field gradient is ramped.

Figure S2. Influence of the RFs amplitudes between the mobility cell and the mass spectrometer

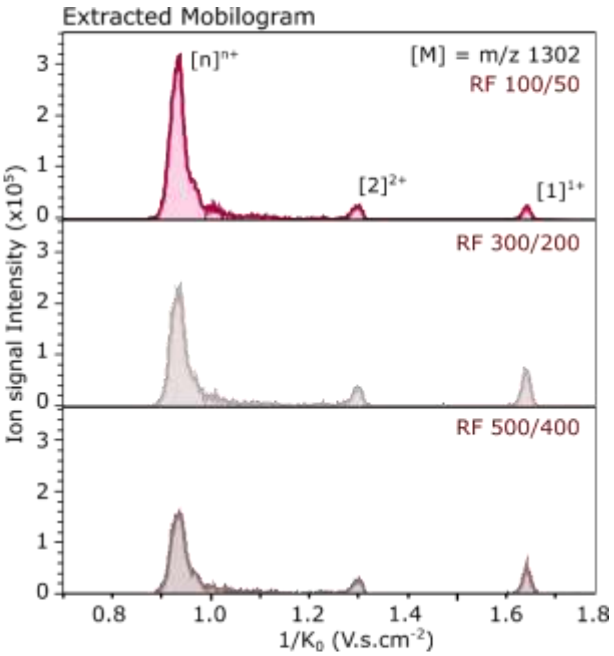

Figure S2: Influence of RF1/RF2 voltages, applied at the second TIMS tunnel and the multipole of the mass spectrometer respectively, on the signal of the extracted reduced mobility spectrum of the transmitted ions m/z 1301.5.

Figure S3. MS/MS spectra of the m/z 651.3 +/- 5 at three different collision energies

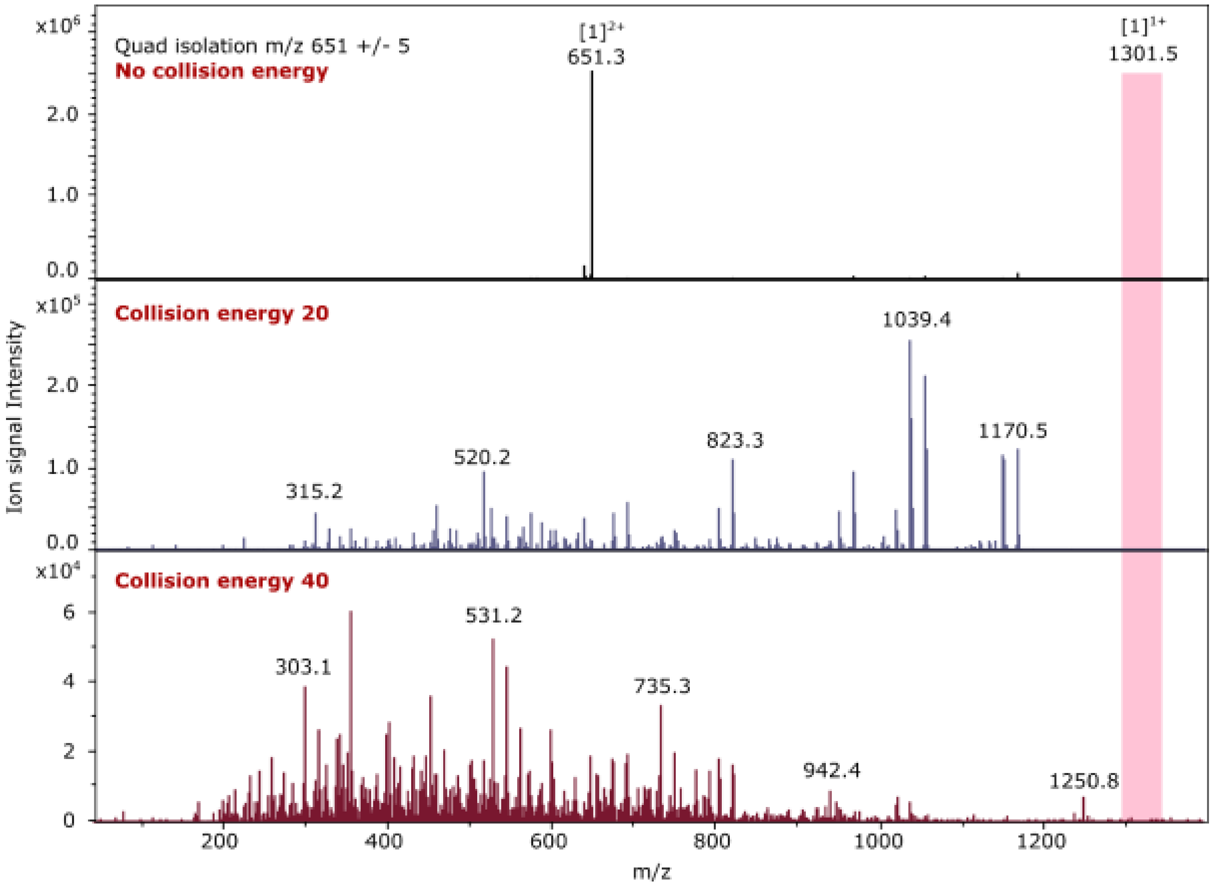

Figure S3: MS/MS spectra of m/z 651.3 +/- 5 with (from top to bottom): no collision energy (black trace) ; collision energy set at 20 (purple trace) and collision energy set at 40 (dark red trace). The pink rectangle highlights the position of m/z 1301.5.

Figure S4. Comparison of mobility spectra from drift tube experiment from the literature and TIMS-ToF experiments of 1301.5 m/z.

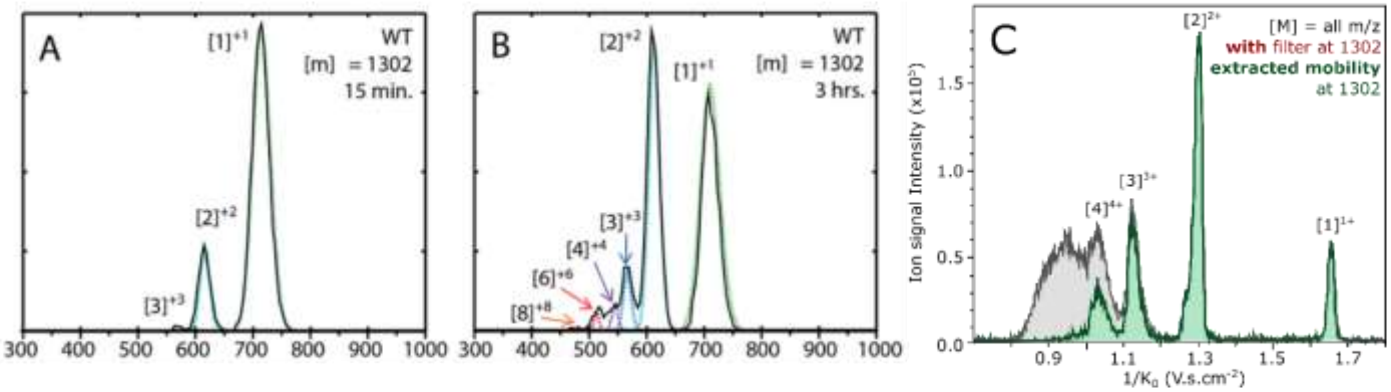

Figure S4: Mobility spectra of m/z 1301.5 from (A and B) drift tube experiments performed by Bowers group and (C) from TIMS experiments with the optimised set of method described in Table S1 with the use of quadrupole filtering set at 1301.5 +/- 5 m/z (green and grey), and the extracted mobility spectrum of the 1301.5 m/z channel in green .

"Here, we compare the experimental results obtained using a drift tube ion mobility mass spectrometer performed by Laos et al. (Fig.S4. A and B) and the trapped ion mobility mass spectrometer performed in this study (Fig.S4. C), on WT peptide from TDP-43 protein. The drift tube experiment (DT) displays measurements for two different incubation times (15 minutes and 3 hours), while the TIMS experiments were generally performed after longer and randomized incubation times. For the DT experiment, we observe the participation of mostly three ions, namely the monomer singly charged $[1]^{1+}$, the dimer doubly charged $[2]^{2+}$ and to a lesser extent the trimer triply charged $[3]^{3+}$, with different ratio depending on the incubation time. The dimer and trimer are not baseline separated, and the signal arising from higher mobility value (e.g. larger oligomers) is not resolved in the drift tube experiments.

TIMS allows to obtain a better separation of the monomeric assemblies present under the same m/z value (m/z 1302). We observe clearly the participation of the same three aggregates, namely, $[1]^{1+}$, $[2]^{2+}$ and $[3]^{3+}$, but also from $[4]^{4+}$ as can be observed in the green spectrum of Figure S4.C. Although similar features are observed, the TIMS has a better separation in mobility, thereby allowing baseline separation between $[2]^{2+}$, $[3]^{3+}$ and $[4]^{4+}$. The signal arising from higher mobility value (e.g. larger oligomers) is not resolved (grey shade), most probably due to fragmentation of higher order clusters in various $[n]^{nz+}$ channels. Moreover,. mass resolution of the TIMS-qToF spectrometer is significantly better, allowing us to use the isotopic distribution (see Fig.2.c) of these ions for unambiguous assignment of the ion mobility peaks."
